# Quantitative Trait Loci and Inter-Organ Partitioning for Essential Metal and Toxic Analogue Accumulation in Barley

**DOI:** 10.1371/journal.pone.0153392

**Published:** 2016-04-14

**Authors:** Stefan Reuscher, Andreas Kolter, Astrid Hoffmann, Klaus Pillen, Ute Krämer

**Affiliations:** 1 Department of Plant Physiology, Ruhr University Bochum, Bochum, Germany; 2 Institute of Agricultural and Nutritional Sciences, Martin-Luther-University Halle-Wittenberg, Halle/Saale, Germany; Julius Kuehn-Institute (JKI), GERMANY

## Abstract

The concentrations of both essential nutrients and chemically similar toxic analogues accumulated in cereal grains have a major impact on the nutritional quality and safety of crops. Naturally occurring genetic diversity can be exploited for the breeding of improved varieties through introgression lines (ILs). In this study, multi-element analysis was conducted on vegetative leaves, senesced flag leaves and mature grains of a set of 54 ILs of the wild ancestral *Hordeum vulgare* ssp. *spontaneum* in the cultivated variety *Hordeum vulgare* ssp. *vulgare* cv. Scarlett. Plants were cultivated on an anthropogenically heavy metal-contaminated soil collected in an agricultural field, thus allowing simultaneous localization of quantitative trait loci (QTL) for the accumulation of both essential nutrients and toxic trace elements in barley as a model cereal crop. For accumulation of the micronutrients Fe and Zn and the interfering toxin Cd, we identified 25, 16 and 5 QTL, respectively. By examining the gene content of the introgressions, we associated QTL with candidate genes based on homology to known metal homeostasis genes of Arabidopsis and rice. Global comparative analyses suggested the preferential remobilization of Cu and Fe, over Cd, from the flag leaf to developing grains. Our data identifies grain micronutrient filling as a regulated and nutrient-specific process, which operates differently from vegetative micronutrient homoeostasis. In summary, this study provides novel QTL for micronutrient accumulation in the presence of toxic analogues and supports a higher degree of metal specificity of trace element partitioning during grain filling in barley than previously reported for other cereals.

## Introduction

Over the millennia, the domestication and breeding of crops has entailed a reduction in genetic diversity accompanying crop improvement that propelled human civilization and population growth. Accordingly, this had the unintended effect of loss of valuable traits that were not selected for, such as pathogen resistances, nutrient efficiencies or some aspects of nutritional value. To overcome this, introgression lines (ILs) can be used today as a source of alleles that positively affect agronomically desirable traits [1,2].

A large proportion of the world population relies primarily on a staple diet for nutrition. The grains of cereal crops, however, are notoriously deficient in both content and bioavailability of the micronutrients zinc (Zn) and iron (Fe), a fact that contributes significantly to global malnutrition. It has been estimated that more than 30% of the world population are suffering from nutritional Fe and Zn deficiencies [3,4]. Enhancing bioavailable micronutrient concentrations in cereal grains, termed bio-fortification, is thus a major goal in current traditional and molecular breeding efforts [5–11]. Instead, grain micronutrient contents have been steadily decreasing in modern cultivated cereal varieties [12]. Moreover, in the light of dwindling mineral resources and increasing fertilizer costs, there is a rising demand for crops with enhanced nutrient efficiency also in areas where soils are deficient in Fe and Zn, which are widespread globally [13].

Industrialization and modern agricultural practices have been introducing unparalleled amounts of potentially toxic compounds into the environment [14]. By virtue of their similarity to inorganic nutrient ions, heavy metals and metalloids, such as cadmium (Cd) and arsenic (As), respectively, can be taken up by plant roots *via* nutrient transporters in the plasma membrane and can move inside plants along nutrient translocation pathways. For example, Cd^2+^ ions have chemical properties similar to the micronutrients Fe^2+^ and Zn^2+^, and AsO_4_^3-^ and AsO_3_^3-^ are phosphate and borate analogues, respectively. Through the combination of direct human intake and accumulation within the food web, the human burden of the toxic nutrient analogue Cd reaches critical levels that have measurable detrimental effects on human health today [15–18]. One future breeding goal is thus to reduce Cd accumulation in cereal grains. Although there is some evidence that plants can discriminate between nutrient and chemically similar toxin ions at some sites of their movement pathways inside the plant, little is known about the precise nature, locations, genetic basis and biochemical and regulatory mechanisms of such discrimination [19].

Natural variation is a promising resource for the recovery of alleles conferring desirable micronutrient-related traits. This present study in barley as a model staple crop aims to explore genetic diversity with respect to its effects on micronutrient and toxic ion accumulation. As a genetically tractable model crop it is closely related to other major cereal crops, such as wheat, rye and oat. Across barley and its ancestral landraces, significant trait variation in metal homeostasis can be expected because soils in the region of historical barley domestication in the Near East are highly Zn-deficient [13]. Moreover, emerging genetic and genomics resources for barley are now beginning to enable molecular genetic approaches towards analyzing the phenotypic consequences of the substantial genetic diversity present among the ancestors of barley [20–25]. Most molecular genetic insights into cereal micronutrient and toxic metal homeostasis have thus far been obtained in rice. However, because of the mostly submerged growth of rice in the presence of the highly bioavailable, ferrous (Fe^II^) oxidation state of iron, there is a need to develop our knowledge of metal nutrition and homeostasis in dryland cereal crops [26].

Here we explore the possibility of selectively increasing grain Zn and Fe concentrations while decreasing the accumulation of the chemically similar toxin Cd through breeding. For this purpose, we cultivated a panel of introgression lines (ILs) in a soil originating from an agricultural field contaminated with low levels of heavy metals. We employed a set of 54 ILs, which were generated by using a wild barley variety from Israel (ISR42-8, *Hordeum vulgare* ssp. *spontaneum*) as a donor and the elite malting cultivar ‘Scarlett’ (*Hordeum vulgare* ssp. *vulgare*) as the recurrent parent [27]. In the past, these introgression lines were successfully used to detect QTL for a variety of agronomically relevant traits, such as pathogen resistance, malting quality, yield and threshability [22,28,29,23,30,31].

To gain a first understanding of the complex suite of uptake and re-localization processes governing grain composition [7], we additionally analyzed element concentrations in one leaf harvested at the juvenile stage and in the senesced flag leaf at maturity. These analyses provided information on primary element accumulation in the vegetative shoot and an estimate of immobilization in, *versus* remobilization from, leaves during grain filling, respectively. Based on these combined datasets, we report the identification of 26 QTL for grain elemental contents. Our data suggest a selective remobilization of the nutrients Fe and Cu from leaves during grain filling, whereas high concentrations of Cd, Ca, Mg and—to a lesser degree—Mn are left behind in the senesced flag leaf, under the growth conditions employed.

## Materials and Methods

### Plant material

A set of 54 introgression lines (ILs; S42-IL101 to 153 and 157) was used together with their recurrent parent Scarlett [32]. Each IL contains at least one chromosomal introgression from the Israeli wild barley accession ‘ISR42-8’ (*Hordeum vulgare ssp*. *spontaneum*), whereas the background genome is consistently derived from the German spring barley cultivar Scarlett (*Hordeum vulgare ssp*. *vulgare*). The chromosomal introgressions in these ILs were mapped previously based on high resolution SNP genotyping analysis [30], and this information was used in the present study.

### Plant growth and harvest

Plants were germinated in petri dishes on wet blotting paper in the dark at room temperature (RT) for 6 d. Seedlings were transferred to pots containing GS90 soil (Einheitserde und Humuswerke, Sinntal-Altengronau, Germany) and cultivated for 5 d before transfer to randomized positions in QPR35 polystyrene trays (Herkuplast-Kubern, Germany) filled with contaminated substrate. Low-level contaminated soil was excavated from the margins of an agricultural field in Northern Germany near Schladen in the floodplain of the river Oker. No written permission was required for this activity at this specific location, because it was conducted in conjunction with a member of the local authority in charge of this site (Lower Saxony *Landesamt für Bergbau*, *Energie und Geologie*, *LBEG*, Hanover, Germany), it involved only a small amount of soil, it did not impinge on the habitats of any endangered or protected species, and the collection site was not covered by any legislation that would have prohibited this activity. For barley cultivation, the soil was mixed with sand (1 vol. part soil + 0.37 vol. parts sand) and osmocote 1½ M fertilizer (0.81 g kg^-1^). Final soil pH was 7.5 ± 0.1 (*n* = 5; for soil elemental composition see S1 Table), determined upon shaking 3 g of soil in 7.5 mL 1 M KCl at 150 rpm overnight, followed by filtering through Whatman No. 1 filter paper. Each of the 35 wells (5 x 7) per tray held one single plant in a volume of approximately 120 ml of substrate. A total of 21 (3 x 7) trays were used to grow all 54 ILs with *n* = 7 replicate plants, with one plant per pot, for each IL. Of the recurrent parent Scarlett seven replicate plants were grown per tray, resulting in *n* = 147 replicate Scarlett plants in total. To avoid positional effects at the margin of the experimental area, all edge positions were occupied by additional Scarlett plants which were not included in the later analysis. Plants were grown in a greenhouse during spring with temperature settings of a minimum of 15°C (night) and a maximum of 20°C (day) and supplemental lighting provided by mercury vapor lamps set to a 16 h light (78 to 138 μmol s^-1^ m^-2^)/8 h dark cycle. Plants were watered from below every two to three days by flooding the greenhouse table briefly. Fifty d post-germination, the third leaf from the top was harvested (designated here: young leaf) and processed for multi-element analysis (BBCH stage 16 to BBCH stage 19). The plants were then allowed to grow to maturity, i.e., to flower, set seeds and dry completely, which corresponds to BBCH stage 97. At maturity, the dry flag leaf (designated here: flag leaf) and all grains were harvested from each plant, allowed to dry in ambient air, and processed for multi-element analysis. Line 113 did not yield any grains.

### Multi-elemental analysis

Leaf tissues were coarsely homogenized using ceramic scissors, and *ca*. 150 mg dry biomass per sample were used for analysis. Grains were heated to 150°C for 1 h, followed by manual removal of husks. Five randomly chosen grains were then pooled and used for each analysis. In a series of preparatory analysis, this was shown to be sufficient for obtaining stable analytical results.

All plant tissues were completely mineralized upon adding 4 mL 65% (v/v) HNO_3_ and 2 mL 30% (v/v) H_2_O_2_ in a microwave-assisted chemical digestion system (MARS5 Express, CEM, USA). The temperature profile was: ramp from RT to 195°C over 20 min, constant at 195°C for 20 min, ramp down to RT over approximately 30 min. After mineralization, each sample was filled up to a final volume of 10 mL with ultrapure water. For the measurement of total element concentrations in soil samples, 0.25 g of air-dried soil, sieved to 2 mm particle size, was mixed gently with 0.75 mL concentrated HNO_3_ (65% w/w) and 2.25 mL concentrated HCl (37% w/w). The soil-acid mix was heated from RT to 160°C in 15 min, held at 160°C for 15 min and cooled down over 45 min in sealed containers in a microwave-assisted chemical digestion system. Samples were filled up to 10 mL final volume with ultrapure water and filtered through Whatman No. 595½ paper before analysis. For the measurement of exchangeable metal concentrations, 10 mL of 0.01 M BaCl_2_ was added to 1 g of dried and sieved soil in a 15-mL screw-cap polypropylene tube, followed by overhead mixing at 150 rpm at RT overnight. Samples were then filtered as described above, followed by the addition of 1 mL 65% HNO_3_ (w/w) before analysis. For the determination of soil pH, 3 g of dried and sieved soil were suspended in 7.5 mL of 0.01 M CaCl_2_ in a 15-mL screw-cap polypropylene tube and shaken at 150 rpm at RT overnight on an overhead shaker. Upon centrifugation at 2000xg and RT for 10 min, pH was determined in the supernatant using a pH electrode (InLab Semi-Micro, Mettler-Toledo, Giessen, Germany) calibrated with pH standards of pH 4, 7 and 10 (Waldeck GmbH & Co. KG, Münster, Germany).

The concentrations of Cd, Zn, Fe, Cu, Mn, Mg and Ca were quantified in plant tissues by inductively-coupled plasma atomic emission spectrometry (ICP-AES) using an ICAPDuo 6500 system (Thermo Scientific, Dreieich, Germany). Samples were supplied to the plasma as an aerosol in Ar gas by a concentric nebulizer. The system was calibrated using “Multi-element standard solution 5” (Sigma-Aldrich, USA) for plant tissue digests, and a concentration series of 5 multi-element standard solutions, pipetted from single-element standard solutions (AAS Standards, Bernd Kraft, Duisburg, Germany), for soil analysis. Performance of the instrument was validated by analyzing certified reference material (IPE No. 547, barley grains) supplied by the Wageningen Evaluating Programs for Analytical Laboratories (Wageningen, Netherlands) every 50^th^ sample and at the beginning and the end of each run of up to 200 plant tissue digests. Soil analysis was validated through measuring digests of NIST 2709a and 2780 (San Joaquin Soil and Hard Rock Mine Waste, LGC Standards, Wesel, Germany) before and after analysis of the soil samples. The measured Cd concentrations, especially in the grains were below the concentration of the lowest calibration solution, but still above blank measurements and above the minimal quantification limit reported by the software of the instrument.

### Statistical analysis

Statistical analyses were carried out with SAS version 9.1 (SAS Institute Inc., Cary, NC, USA). Genetic correlations between trait values were calculated using the least squares means (LSMEANS) for each of the 54 ILs and Scarlett averaged across all replicates. Broad-sense heritability was calculated as *H*^*2*^ = *V*_*G*_/ [*V*_*G*_ + *V*_*R*_/*n*], applying the SAS procedure VARCOMP. The terms *V*_*G*_ and *V*_*R*_ and *n* represent the genotypic and error variance components, and the number of replicate plants per genotype, respectively. The LSMEANS for each IL and Scarlett were computed with a mixed linear model (MLM, procedure MIXED) and used for further analysis [33,29]. The MLM procedure included a two-factorial analysis of variance using the genotype as a fixed factor with 55 levels (54 ILs and Scarlett) and the row and column of the respective plant across the entire cultivation area as a random factor with 13 and 47 levels, respectively. This was conducted on grain element concentrations as the major focus of this study, and separately for element concentrations in young leaf and flag leaf, respectively, for the identification of QTL (see below). We also conducted a full model analysis, which additionally included tissue as a fixed factor, and highlighted major differences between tissues (see ANOVA in S1 Dataset). Median values shown in S1 Fig, by contrast, were calculated without taking into account positional effects. Significant differences between LSMEANS were revealed by Dunnett’s multiple comparison test [34] using Scarlett as the control followed by a false-discovery rate analysis (procedure MULTTEST with the FDR option, which implements the linear step-up method of Benjamini and Hochberg [35]). When concentrations of an element differed significantly from the recurrent parent Scarlett in a given IL (corrected *P* value < 0.05; *FDR_P*), it was considered to carry a so far unknown trait-associated gene that caused the effect. This unknown gene is termed quantitative trait locus (QTL) since it acts quantitatively on element concentration in ILs. The chromosomal position of the QTL is restricted to the described extension of the wild barley introgression [30]. If two or more ILs with overlapping wild barley introgression share the same QTL effect, then the QTL’s chromosomal position is concluded to be constrained to the overlapping segment of the introgressions.

There is a possibility that one QTL identified here contains several causal loci that have different effects. In addition to the longest unique, so-called “target”, introgression, most ILs contain secondary, smaller additional introgressions, and an identified QTL according to the use of the term in this study would then comprise all introgressions of a given IL, which are documented. Moreover, a given IL may contain additional undetected introgressions, which should generally be only small in size [30].

## Results

During cultivation in the heavy metal-containing agricultural soil (see Materials and Methods) neither the set of 54 ILs nor the recurrent parent Scarlett showed any symptoms of metal toxicity. We attribute this to high soil exchangeable Ca and Fe concentrations, a comparably high soil pH, moderate Zn and low exchangeable soil Cd concentrations available to plant roots for uptake (S1 Table). Similarly, the crops observed to grow on this soil in the field were free of symptoms of metal toxicity (U. Krämer, unpublished observation). The primary focus of this study was on the concentrations of the essential micronutrients Zn and Fe, as well as of the toxic heavy metal Cd, in grains of barley plants at maturity. We additionally monitored the concentrations of the micronutrients Cu and Mn and the macronutrients Ca and Mg because of their known physiological interactions with Cd, Fe and Zn in plants. In order to approximate the elemental composition of a leaf dedicated to photosynthesis and carbon assimilation, the third-youngest leaf was harvested from plants at the five-leaf stage (young leaf). Finally, the senesced flag leaf was harvested for multi-element analysis alongside the grains after the plants had completed their life cycle.

### Metal specificity of elemental profiles across different tissues of barley plants

**Fig 1:**
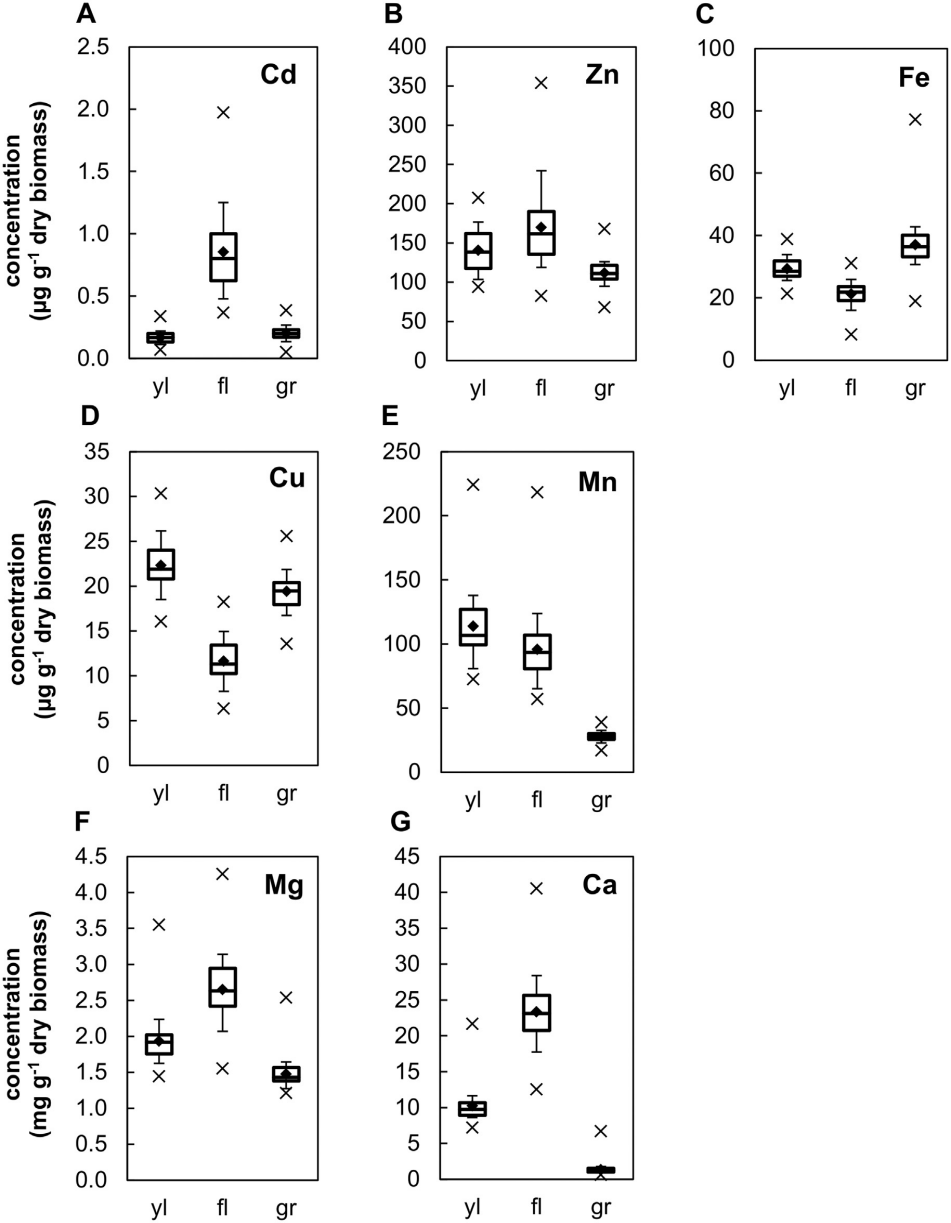
Element accumulation in tissues of barley introgression lines. Concentrations of (A) Cd, (B) Zn, (C) Fe, (D) Cu, (E) Mn, (F) Mg, and (G) Ca are shown in boxplots summarizing least-square means of the 54 ILs and the recurrent parent Scarlett, for young leaf (yl), senesced flag leaf (fl) and grains at maturity (gr) of plants grown on a contaminated soil. Diamonds represent arithmetic means calculated from all 55 genotypes, 25-to-50-to-75 and 10-to-90 percentiles are represented by boxes and whiskers, respectively, and minimum and maximum values are shown as cross symbols. Plants were cultivated in a greenhouse on a soil collected in a heavy-metal contaminated agricultural field, with harvest of yl at the five-leaf stage and harvest of fl and gr at maturity. Note that concentrations of the macronutrients Mg (F) and Ca (G) are plotted on a different scale. Each measurement was conducted on pooled tissues collected from one plant, with least-square means calculated from three to seven replicate plants for each IL and from 144 replicate plants for Scarlett, respectively (see also Materials and Methods).

Out of all elements quantified in this study, tissue concentrations of the non-essential, toxic transition metal Cd were the lowest. Grain Cd concentrations ranged between an observed minimum of 0.05 μg g^-1^ dry biomass (least-square mean) in IL107 and a maximum of 0.39 μg g^-1^ in IL115, with 0.23 μg g^-1^ in the recurrent parent Scarlett (Fig 1A, S1 Dataset, refer to S2 Table for *H*^*2*^, see also Table 1 and text below). Overall, Cd concentrations were *ca*. four- to five-fold higher in the flag leaf (averaging 0.85 μg g^-1^) than in grains (averaging 0.20 μg g^-1^) or in the young leaf (averaging 0.17 μg g^-1^)(Fig 1A). These data indicate that barley is capable of immobilizing Cd in the flag leaf during grain filling, thus apparently restricting the transfer of Cd from flag leaf into grains. Flag leaf-to-grain Cd ratios of Scarlett approximated global averages, and the ratios of individual ILs ranged between 1.9 and 19. Our results indicate major variation among ILs in grain Cd accumulation and Cd partitioning.

**Table 1:** QTL for Cd, Zn and Fe concentrations in mature grains. Magnitude of effect of each QTL is expressed as percentage of the concentration found in the parent genotype Scarlett (% of Sc).

IL Id.	Element	% of Sc	*P* value	Target introgression Chromosome	Target introgression Position (cM)^1^	Additional introgressions Position (cM)^1^,^2^
IL157	Zn	150	< 0.001	1H	64.79–90.92	2H 20.11–22.35
IL107	Cd	24	< 0.01	2H	34.31–66.78	2H 158.39–163.20
IL107	Zn	61	< 0.001		34.31–66.78	2H 158.39–163.20
IL108	Fe	62	< 0.01		34.31–104.81	2H 158.39–161.08 *h*
IL115	Cd	170	< 0.01	3H	204.48–255.13	6H 137.48–147.51
IL115	Zn	127	< 0.01		204.48–255.13	6H 137.48–147.51
IL146	Fe	206	< 0.001	4H	83.58–119.06	-
IL123	Fe	51	< 0.01		128.85–172.32	2H 207.22–213.08, 4H 61.15–74.11, 4H 83.58

^1^ [30];

^2^ *h* following the genomic position indicates a hemizygous introgression.

Concentrations of the essential micronutrient Zn were more than two orders of magnitude higher than those of Cd in all analyzed tissues. Grain Zn concentrations were between 68 μg g^-1^ in IL107 and 170 μg g^-1^ in IL157, again with an intermediate concentration of 110 μg g^-1^ in Scarlett (Fig 1B, S1 Dataset, S2 Table, Table 1). In contrast to Cd, average Zn concentrations across genotypes were of a similar magnitude in all three tissues (young leaf: 140 μg g^-1^; flag leaf: 170 μg g^-1^, grains: 110 μg g^-1^; Fig 1B). Thus, Zn partitioning was different from Cd partitioning in barley, with proportionally less Zn immobilization in the flag leaf or more Zn transfer into grains. This occurred despite the presence of an excess of Zn as well as an elevated Cd concentration in the soil. Compared to other tissues, flag leaves of the different ILs accumulated between 0.6-fold and 3.7-fold Zn concentrations, again with Scarlett positioned centrally in between extreme ratios. Thus, variation in flag leaf-to-grain partitioning of Zn is large among ILs, but considerably smaller than for Cd.

Grain concentrations of the essential micronutrient Fe ranged between a minimum of 19 μg g^-1^ in IL123 and a maximum of 77 μg g^-1^ in grains of IL146 (Fig 1C, S2 Table, Table 1). Grains of Scarlett contained 37 μg g^-1^ Fe. More similar to Zn than to Cd, across all lines tissue concentrations averaged within a narrow range between 29 μg g^-1^ in the young leaf, 21 μg g^-1^ in the senesced flag leaf, and 37 μg g^-1^ in grains. Fe was the only element for which we observed higher mean concentrations in the grains than in either the young leaf or the flag leaf. This suggests that Fe partitioning to the grains was generally favored in barley ILs, with 1.1 to 5-fold higher Fe concentrations in grains than in the flag leaf (Scarlett: 1.8) and ratios of grain to young leaf of between 0.6 and 2.2 (Scarlett: 1.1). Overall, variation in the accumulation and partitioning of Fe among ILs was slightly larger than for Zn.

**Fig 2:**
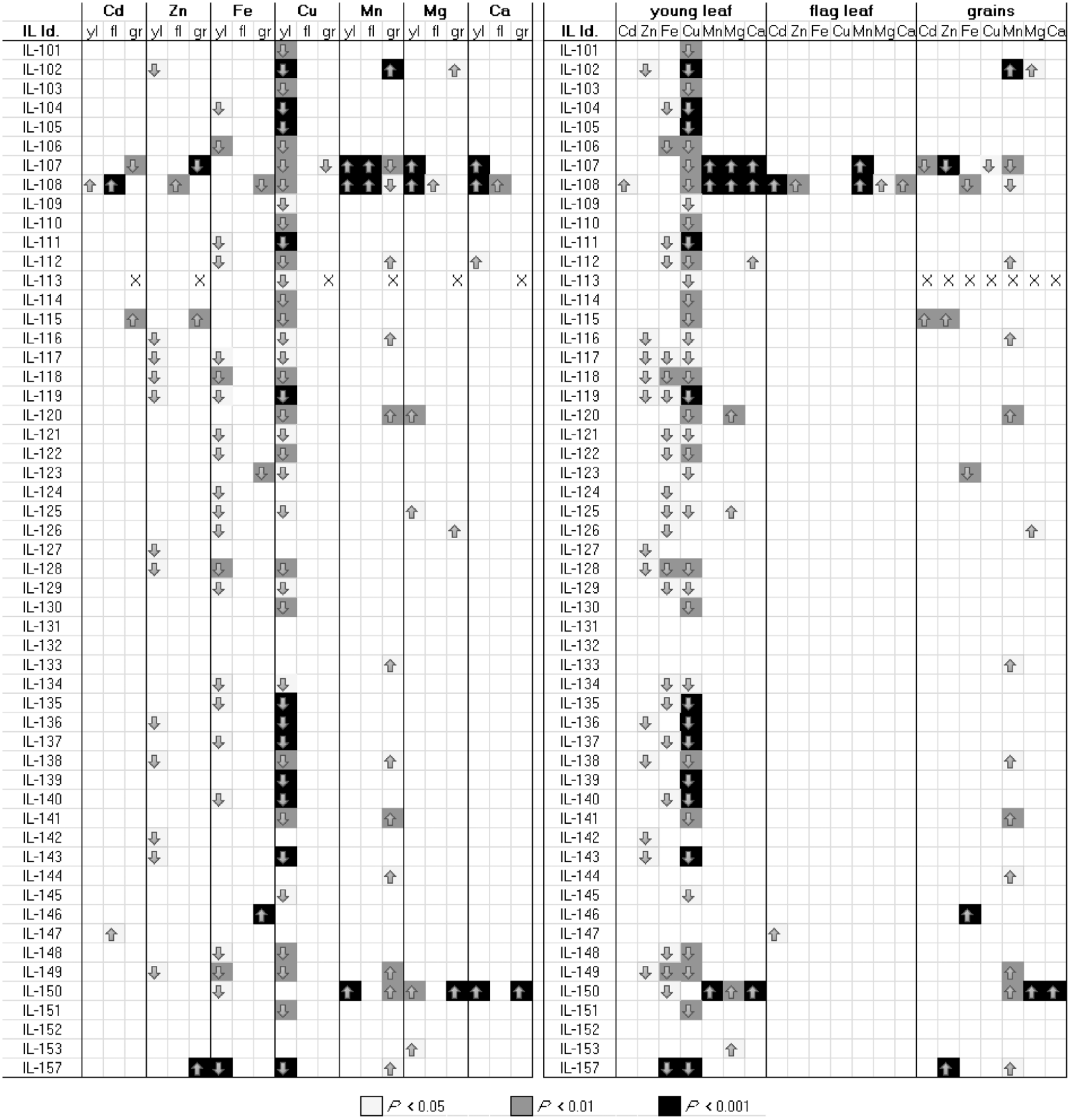
Overview of all QTL detected in all tissues. Shown is an overview of all statistically significant effects on concentrations of Cd, Zn, Fe, Cu, Mn, Mg and Ca in the young leaf (yl), flag leaf (fl) or the grains (gr). The same dataset is shown twice, (A) grouped by element, and (B) grouped by tissue. Arrows show the direction of the effect relative to the recurrent parent Scarlett, background color of boxes show *P* values as a measure of statistical significance (FDR_P; see legend). Genotype effects were detected using a mixed model approach as described in materials and methods (main text). Statistical significance was tested using Dunnett’s test, followed by FDR analysis.

Grain concentrations of the essential micronutrient Cu ranged between a minimum of 14 μg g^-1^ in IL107 and a maximum of 26 μg g^-1^ in IL150 that was not significantly above the average of 20 μg g^-1^ in Scarlett (Figs 1D and 2, S1 Dataset). Average Cu concentrations differed only moderately between tissues (young leaf: 22; flag leaf: 12, grains: 19 μg g^-1^) and were lowest in the flag leaf, thus yielding an overall profile most similar to the profile observed for Fe. Grain Mn concentrations ranged from a minimum of 17 μg g^-1^ in IL107 to a maximum of 39 μg g^-1^ in IL102, again with intermediate Mn concentrations in grains of Scarlett of 26 μg g^-1^ (Figs 1E and 2). In contrast to the other essential micronutrients (Zn, Fe, Cu), average concentrations of Mn in the grains were about four-fold lower than in the leaf tissues (young leaf: 110 μg g^-1^, flag leaf: 96 μg g^-1^, compared to grain: 28 μg g^-1^). Ratios of flag leaf to grain concentrations were between 11.6 in IL108, 3.5 in Scarlett and 1.9 in IL124. The profile was thus intermediate between the profiles for Cd and Zn and suggested that under the given conditions of high Mn supply in the soil, barley plants were able to restrict Mn accumulation in the grain. Instead, Mn concentrations in the young leaf reached high levels. This may reflect the prominent use of Mn in the photosynthetic apparatus.

Grain concentrations of the macronutrients Mg and Ca were similar and generally in the low mg g^-1^ range (Fig 1F and 1G). As noted above also for Cd, Zn Cu and Mn, IL107 was among the lines exhibiting the lowest grain concentrations of both Mg (1.2 mg g^-1^) and Ca (0.67 mg g^-1^), but not significantly below Scarlett for these macronutrients. By contrast, Fe concentrations in grains of IL107 (34 μg g^-1^) were close to the mean of all genotypes (37 μg g^-1^) (S1 Dataset, Fig 2). Highest grain concentrations for both Mg (2.5 mg g^-1^) and Ca (6.7 mg g^-1^) were found in IL150, which was also high in Mn (36 μg g^-1^) (Fig 2), as well as comparably, but not statistically significantly, high in Cu (see above) and Cd (0.33 μg g^-1^), whereas it contained only moderately elevated Fe (47 μg g^-1^)(S1 Dataset). Average Ca concentrations were up to eight times lower in grains than in the young leaf (young leaf: 10, flag leaf: 23, compared to grains: 1.4 mg g^-1^). Ratios of Ca concentration in grains relative to flag leaf were between 40.7 in IL108 and 3.3 in IL150. The overall relative profile of Ca concentrations across different tissues was most similar to that of Cd (Fig 1), whereas the overall profile of Mg concentrations was more similar to the profile of Zn concentrations.

Proportionally, the largest range of variation among ILs was observed for grain Ca concentrations (10-fold higher mean in maximum IL than in minimum IL), followed by Cd (7-fold) and Fe (4-fold), with similar and lower variation for Zn, Mg, Mn and Cu (all around 2-fold).

### QTL analysis

Element concentrations in the young leaf, the flag leaf and in grains of the 54 ILs and the recurrent parent Scarlett were subjected to a genotype (IL) by phenotype association study. The average broad-sense heritability *H*^2^ of all traits, i.e. element concentrations, was 75% ± 15% (arithmetic mean ± SD), indicating that a high proportion of the observed phenotypic variation is a result of genetic variation (all *H*^2^ values in S2 Table). A two-factorial mixed-model analysis identified a total of 122 statistically significant effects in different tissues (Fig 2, S3 Table). For simplicity, we address each significant effect for an element as one QTL, which includes both the target introgression and additional secondary introgressions of the IL in which the effect was detected, and these can comprise several contributing causative genes (see Materials and Methods). Out of these, 41 QTL were associated with higher element concentrations in the ILs compared to Scarlett, whereas 81 QTL were associated with lower element concentrations (*P* < 0.05). QTL were found for each element in each tissue, with the exception of Fe and Cu in the flag leaf. Here, we will focus on the elements of central interest in the current work, namely Cd, Zn and Fe.

For Cd, a total of five QTL were identified based on four different ILs. Grain Cd concentrations differed significantly from those found in Scarlett (Sc) in IL107 (24% of Sc; *P* < 0.01) and IL115 (170% of Sc; *P* < 0.01; Table 1). IL108 showed elevated Cd concentrations in both the flag leaf (230% of Sc; *P* < 0.001; S4 Table) and the young leaf (195% of Sc; *P* < 0.01; S5 Table). IL147 showed elevated Cd concentrations in the flag leaf (200% of Sc; *P* < 0.05) when compared to Scarlett (S4 Table). IL107 (2H) is thus of major interest in relation to the desirable breeding goal of reducing grain Cd accumulation.

A total of 16 QTL were found for Zn. QTL for grain Zn concentrations were detected in IL107 (61% of Sc; *P* < 0.01), IL115 (127% of Sc; *P* < 0.01) and IL157 (150% of Sc; *P* < 0.001; see Table 1). One QTL found in IL108 was found to affect Zn concentration in the flag leaf (179% of Sc, *P* < 0.01; see S4 Table). As many as 12 QTL were identified to influence Zn accumulation in young leaf (ILs 102, 143, 142 (1H), 116–119 (4H), 127 (5H), 128, 149 (6H), 136, 138 (7H); see S5 Table). In relation to the breeding goal of decreasing grain Cd accumulation without simultaneously decreasing grain Zn accumulation, the QTL detected in IL107 was associated with strongly reduced grain Cd and a moderate reduction in grain Zn concentrations. There was only one QTL associated with enhanced grain Zn accumulation (IL157), which was found not to significantly affect grain Cd accumulation in a similar manner. The introgressed donor segment in IL157 (1H) is thus of major interest as a potential target in nutrient-specific Zn biofortification.

Out of a total of 25 QTL detected to affect Fe concentrations, three QTL were associated with altered grain Fe concentration relative to Scarlett (IL108, 62% of Sc, *P* < 0.01; IL123, 51% of Sc, *P* < 0.01; IL146, 206% of Sc, *P* < 0.001; see Table 1). No QTL for Fe concentration in the senescent flag leaf were identified. A total of 22 QTL were found to affect Fe concentrations in young leaf (ILs 104, 157 (1H), 106, 111, 112, 140 (2H), 117–119, 121, 124 (4H), 125, 126 (5H), 148, 128, 129, 149, 150, 122 (6H), 134, 135, 137 (7H); see S5 Table). Thus, IL146 (4H) exhibited remarkably high grain Fe accumulation, which was not accompanied by an accumulation of Cd or Zn. The introgression in this line is thus of major interest in Fe biofortification.

### Differential and selective remobilization of Fe, Cu and Mn from the flag leaf during grain filling

**Fig 3:**
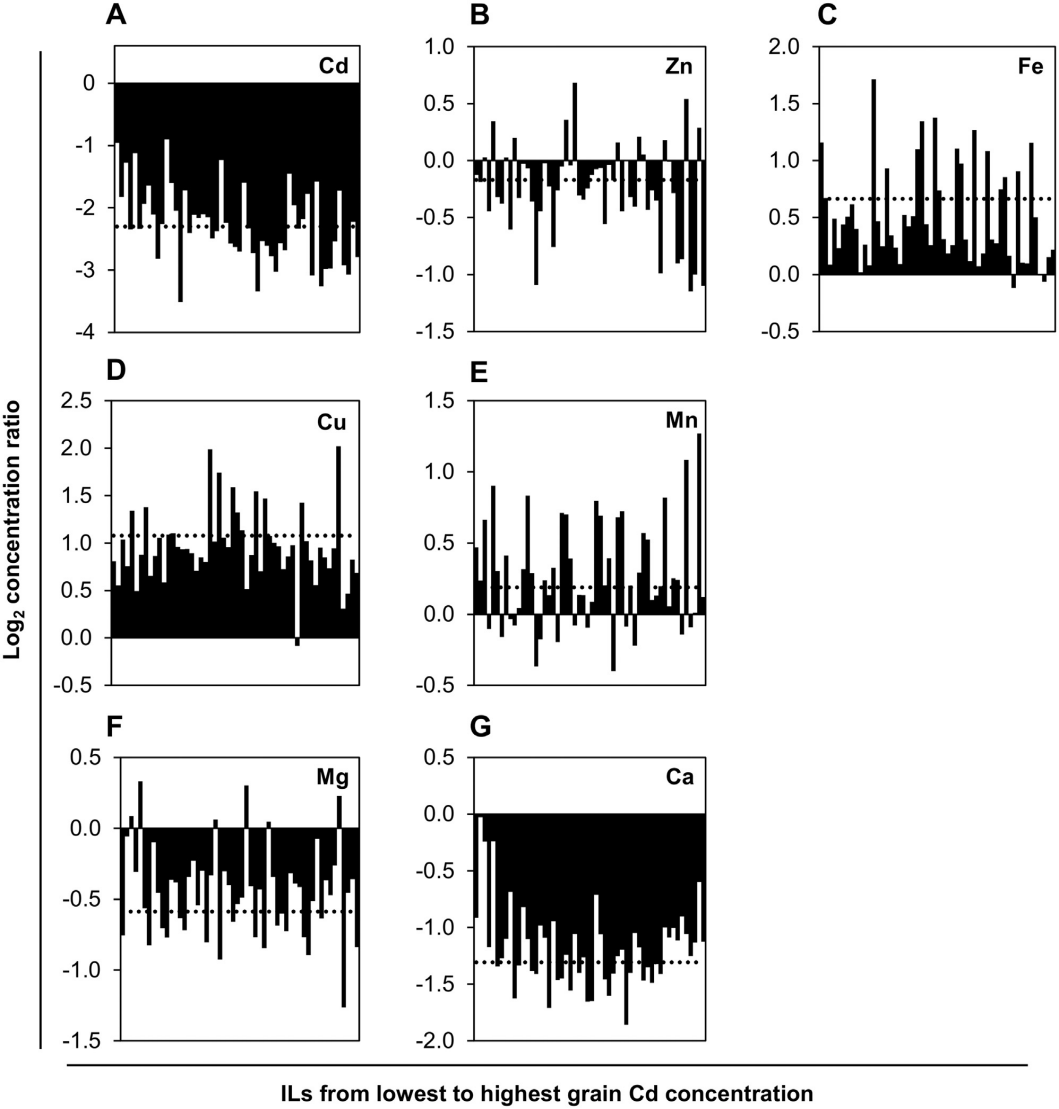
Element accumulation in young leaf relative to flag leaf of 54 barley introgression lines. Shown are Log_2_ ratios of element concentrations in the young leaf *versus* the flag leaf for (A) Cd, (B) Zn, (C) Fe, (D) Cu, (E) Mn, (F) Mg, and (G) Ca. Each bar represents the least-square mean of one IL (*n* = 3 to 7), with ILs sorted by ascending grain Cd concentration from left to right. Dashed horizontal lines indicate the position of the least-square mean of the recurrent parent Scarlett (*n* = 144). Measurements were from the same experiment as shown in Fig 1.

During grain filling, nutrients are delivered to the grain. Nutrients can enter grains from two alternative sources: root uptake from the soil solution followed by root-to-shoot transfer, and remobilization from senescing leaves, in particular the flag leaf [1,7]. Both of these pathways are likely to be specific at least for groups of chemically similar elements, and their contributions might vary in nature dependent on the genotype and the nutrient and metal status of the plant throughout its life cycle and until grain-filling. To approximate the degree of remobilization of nutrients from the flag leaf during grain filling, we compared concentrations remaining in the fully senesced flag leaf to those measured in the young leaf. Across all genotypes, the non-essential, toxic Cd appeared to be remobilized from the flag leaf to an even lesser degree than the two macronutrients Ca and Mg, as indicated by far higher Cd concentrations in the senesced flag leaf compared to the young leaf (Fig 3). By comparison, the essential micronutrients Fe, Cu and Mn seemed to be remobilized from the flag leaf into the grains to a larger degree, as suggested by lower concentrations in the senesced flag leaf compared to the young leaf. The data for Zn suggested an intermediate degree of remobilization from flag leaf.

There was a negative correlation between grain Cd concentration and the ratio of Cd concentrations in young leaf *vs*. flag leaf (*r* = -0.39; *P* < 0.01; see Fig 3A, compare left side with right side of diagram). Moreover, grain Cd concentrations were positively correlated with flag leaf Cd concentrations (*r* = 0.5; *P* < 0.001; data not shown). Thus, a more efficient remobilization of Cd from the flag leaf does not make a predominant contribution to grain Cd accumulation among ILs. There were no correlations between concentrations in mature grains and the young leaf for any of the analyzed elements (S2 Fig).

### Relationships between the concentrations of different elements

**Fig 4:**
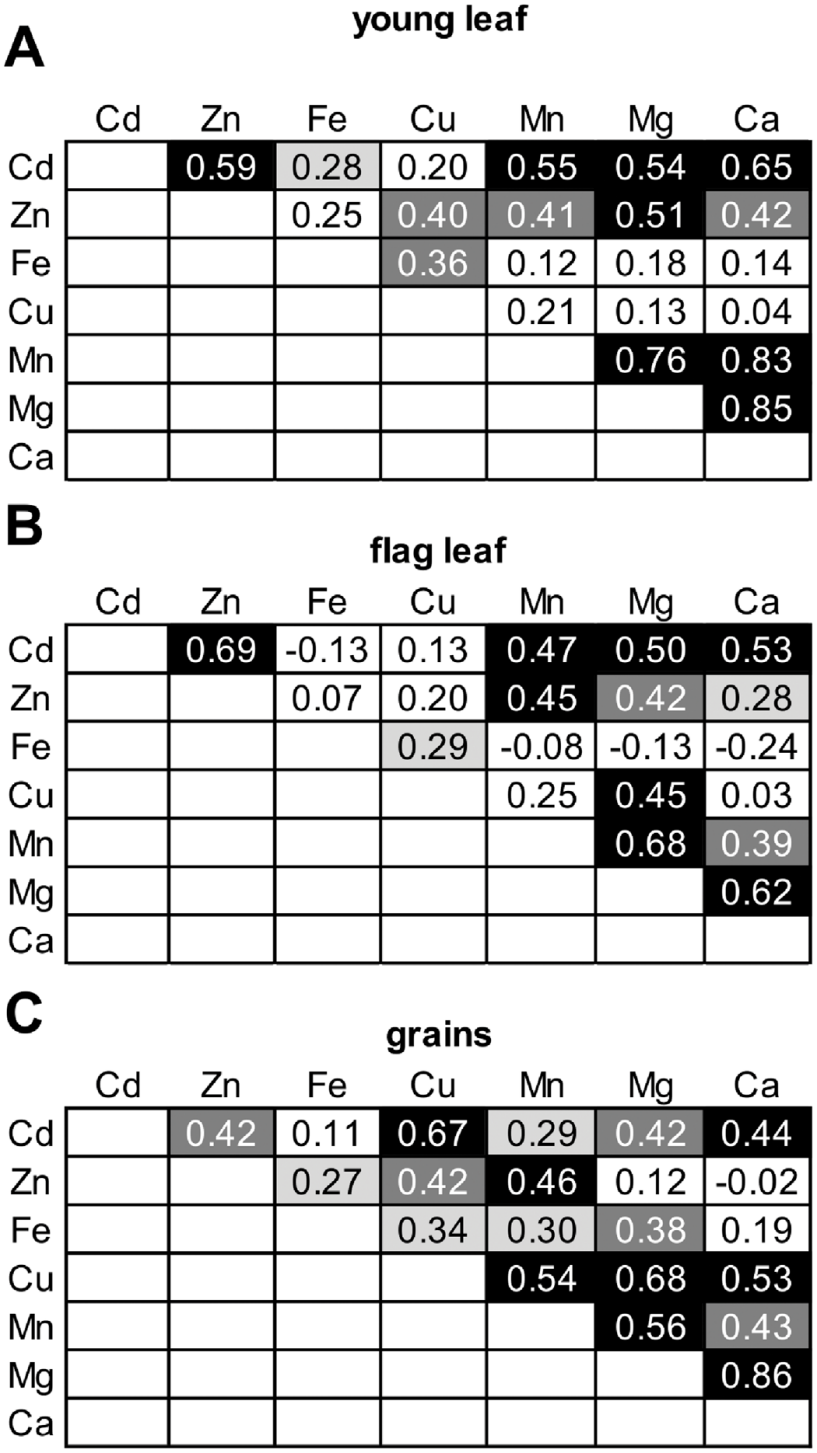
Between-element correlations of concentrations in tissues of barley ILs. Pearson correlation coefficients (*r*) are given for (A) the young leaf, (B) the flag leaf and (C) grains. Values are based on least-square means of 54 ILs and the recurrent parent Scarlett. Correlations exhibiting *r* > |0.27|, |0.35| and |0.44| are statistically significant at *P* < 0.05 (light grey), 0.01 (dark grey) and 0.001 (black), respectively, according to a two-tailed distribution and *n* = 55 observations. Measurements were from the same experiment as shown in Fig 1.

Based on previously established knowledge on the uptake and movement of Zn, Fe and Cd in plants, we expected correlations among the concentrations of Cd, Zn and Fe. A statistically significant correlation between Cd and Fe (*r* = 0.28, *P* < 0.05) was found only in the young leaf (Fig 4). As expected, correlations between Cd and Zn concentrations were found in the young leaf (*r* = 0.59, *P* < 0.0001) and the flag leaf (*r* = 0.69, *P* < 0.0001). Whereas the correlation between grain Cd and Zn concentrations was less pronounced (*r* = 0.42, *P* < 0.01), Cd concentrations were correlated with Cu concentrations in grains (*r* = 0.67, P < 0.0001), but in none of the other tissues.

Correlations were observed between concentrations of the macronutrients Mg and Ca in grains (*r* = 0.86, *P* < 0.0001) and in the young leaf (*r* = 0.85, *P* < 0.0001), indicating that the pathways of movement or the physiological parameters delimiting the capacity for accumulation might be largely shared between these cations (Fig 4). Furthermore, Mn concentrations correlated with those of Mg and Ca in the young leaf (Mg: *r* = 0.76; Ca: *r* = 0.83, both *P* < 0.0001). This is unexpected since Mn is a micronutrient, and its concentrations were thus expected rather to correlate with Zn or Fe concentrations. Possibly, when Mn is overly abundant in the soil, as was the case in our study, the processes that determine Mg and Ca accumulation in leaves constitute a more important factor influencing Mn accumulation than the pathways of movement of Zn or Fe. We found a far less pronounced correlation of grain Mn with grain Mg and Ca concentrations (Mg: *r* = 0.56, *P* < 0.0001; Ca: *r* = 0.43, both *P* < 0.01), which suggests that the control of import into the developing grains operates somewhat differently. This is supported by the much lower extent of variation of Mn concentrations in the grains compared to the young leaf (see Fig 1).

Correlations were also detected between Cd and Mn, Mg and Ca concentrations, respectively, most evidently in young leaf (Mn: *r* = 0.55, *P* < 0.0001; Mg: *r* = 0.54, *P* < 0.0001; Ca: *r* = 0.65, *P* < 0.0001) and flag leaf (Mn: *r* = 0.47, *P* < 0.001; Mg: *r* = 0.50, *P* < 0.0001; Ca: *r* = 0.53, *P* < 0.0001) and less pronounced in grains (Mn: *r* = 0.29, *P* < 0.05; Mg: *r* = 0.42, *P* < 0.01; Ca: *r* = 0.44, *P* < 0.01).

In summary, these observations suggested that the pathways that determine Ca, Zn, Mn and Mg accumulation all contribute to Cd accumulation in young leaf. However, by comparison the influence of these pathways on Cd accumulation in the grain appeared to be reduced, suggesting that distinct and more ion-selective mechanisms operate during grain filling. Specifically during grain filling, Cd might at least partly share a pathway or binding ligand governing Cu movement (see Fig 4C).

## Discussion

Cereal grains are the main staple diet for a large proportion of the global population, and thus classical bio-fortification strategies aim at increasing the concentrations and bioavailability of essential trace elements of cereal grains [36,37]. An advanced goal of crop breeding, however, is to generate cultivars that feature adequate micronutrient concentrations, but simultaneously exclude chemically similar, hazardous analogues that are widespread as soil contaminants in low, but relevant concentrations from fertilizer or sewage sludge application, or from industrial sources [38]. The chemical properties of the essential micronutrients Zn and Fe, in particular, are similar to those of the toxic trace element Cd, and thus these elements largely share common membrane transport and long-distance translocation pathways in plant [39]. Here we undertook a study in barley as a model cereal, because metal homeostasis is expected to operate differently from rice, because gene identification remains more challenging in the closely related hexaploid wheat, and because the ancestral barley landraces originate from a region rich in Zn-deficient soils. QTL were identified for elemental concentrations in three different tissues of plants grown on an agricultural soil contaminated with low levels of heavy metals. Our study is thus unique in being able to compare element accumulation between different tissues. Moreover, by challenging plants with an agricultural soil containing elevated, but non-toxic heavy metal concentrations, we expect to identify QTL that are relevant in contaminated fields.

In a population of 54 ILs, an overall smaller degree of variation in element concentrations of grains, when compared to young leaf and, in particular, flag leaf tissues suggested that the control of element accumulation is tightest in the grains (Fig 1, S1 Fig). By comparison to grains and the young leaf, the senesced flag leaf was highly enriched in Cd, and moderately enriched in Ca and Mg, suggesting an immobilization of these elements in the flag leaf during the grain filling process. By comparison to the flag leaf, grains were generally enriched in Fe and Cu, whereas Mn concentrations were substantially lower in grains than in the young leaf and the flag leaf. The range of variation among ILs was generally considerably larger than among replicates of the recurrent parent Scarlett, indicating that the introgressions increased the range of phenotypic variation, as expected (S1 Fig), with the exception of young leaf Cd concentrations.

Fe was the only nutrient element for which concentrations in the young leaf were in the deficiency range, i.e., below 72 μg g^-1^ generally considered the critical Fe deficiency concentration in plants [40]. In the soil used for cultivation, however, amount and availability of Fe were not low. It is known that elevated soil heavy metal concentrations interfere with Fe homeostasis so that the plants may have experienced an as yet asymptomatic, deficient physiological Fe status under the growth conditions employed here [19]. Average concentrations of Fe were at the low end, Ca, Mn and Mg were normal to slightly elevated, whereas Cu and Zn concentrations were two- to three-fold above the range of concentrations normally found in the barley grain (Fig 1) [41].

### Identification of candidate genes

The experimental design to locate QTL in our introgression library S42-IL can be considered a multiple case-control experiment. Thus, a significant phenotypic deviation of an IL from the control Scarlett may indicate the presence of a hidden gene, located on the introgressed segment of the IL, which is causing the QTL effect. We applied a mixed model-based Dunnett test, including an FDR adjustment for multiple testing, to identify QTL and to avoid detection of false positive QTL effects. In contrast, applying a classical ANOVA or composite interval mapping (CIM) to our data would often result in a biased testing of up to 53 ILs carrying the Scarlett allele against a single IL carrying the exotic donor allele at a particular SNP locus [42,43].

The number of 122 QTL identified among 21 traits studied by multi-element analysis may indicate that only reliable QTL are detected in our study. Nevertheless, follow-up validation experiments with segregating high-resolution offspring of the studied ILs are advisable to validate the identity of the located QTL [30].

To analyze the gene content of the introgressions, the published gene order of barley [22,24] was used in combination with the most recent available genotyping data from the S42-IL collection [30]. Based on these data the genes originating from the donor parent (the hypothesized gene content of the introgressions) were compiled for each IL, taking into account all introgressed segments for each IL. The introgressed gene content of selected ILs was then screened for genes annotated as “metal.handling” or “transport.metal” based on homology to Arabidopsis or rice by the MAPMAN ontology [44]. Additionally, the lists were also manually browsed for genes with known or putative functions in metal homeostasis (S2 Dataset). Our initial focus was on those QTL that were supported by overlapping introgressions of different ILs (e.g. IL117 to 119).

Our QTL analysis highlighted IL107 in particular, in which grain Cd and Zn concentrations were reduced to 24% and 61%, respectively, of those in Scarlett. By contrast, in IL108 that carries an overlapping introgression, grain Cd concentrations were 19% higher than in Scarlett and grain Zn concentrations were only about 15% lower than in Scarlett (see Table 1). Thus, the portion of the introgression that is present in IL107, but absent in IL108, is likely to reduce grain Cd accumulation. A candidate gene in this region encodes a member of the Zinc-regulated transporter Iron-regulated transporter Protein (ZIP) family of divalent transition metal cation transporters, and exhibits highest sequence similarity to *A*. *thaliana ZIP1* ([45]; Table 2). Based on the functional complementation of a yeast mutant and its transcriptional upregulation in response to Zn deficiency, *A*. *thaliana* ZIP1 was proposed to act as plasma membrane Zn^2+^ uptake system. The second candidate gene in this region is predicted to encode a member of the YSL (Yellow Stripe-Like) protein family of the oligopeptide transporter superfamily with similarity to *A*. *thaliana YSL2*, and high similarity to Brachypodium YSL17 as well as rice YSL7 and YSL17 in a grass-specific clade out of four clades of the YSL protein family [46]. Membrane transporters of this protein family were described to mediate the transport of transition metal complexes of nicotianamine or phytosiderophore ligands into the cytoplasm, with roles in root micronutrient uptake and in metal partitioning within the plant and within individual cells.

**Table 2:** Candidate genes identified in selected ILs based on either shared or unique segments of introgressions.

Trait(s)	effect(% of Sc)	IL Id(s).	Chromosome	AGI code or rice locus	Gene name	Description
Zn young leaf	60.24c	117–119	4 H	AT1G47240	NRAMP2	Natural resistance-associated macrophage protein 2
Fe young leaf	77.52			AT5G13750	ZIFL1	Zinc induced facilitator-like 1
Fe young leaf	73.25	104, 157	1H	AT5G27690		Heavy metal transport/detoxification protein domain containing protein
				AT2G30080	ZIP6	Zrt/Irt-like protein 6
				AT1G16380	CHX1	Cation/H^+^ exchanger 1
				AT2G23240	MT4B	Metallothionein 4B
				AT3G53720	CHX20	Cation/H^+^ exchanger 20
				Os05g0368600		Heavy metal transport/detoxification protein domain containing protein
Cd grains	23.51	107	2H	AT5G24380	YSL2	Yellow stripe-like 2
Zn grains	60.68			Os04g0613000	-	*A*. *thaliana* ZIP1-like
Cd grains	169.66	115	3H, 6H	AT5G59520	ZIP2	Zrt/Irt-like protein 2
Zn grains	126.70			AT1G51610	MTP7	Metal tolerance protein 7
				AT3G58060	MTP8	Metal tolerance protein 8
				Os02g0775100	MTP3	Metal tolerance protein 3

Shown are selected QTL with their effect strength (given as percentage of the concentration found in the parent genotype Scarlett) and candidate genes based on homology to Arabidopsis or rice found in the (overlapping/unique) introgressions of the indicated ILs.

IL157 was distinctive by high concentrations of Zn, but not of the toxic element Cd, in grains. The segment unique to IL157 is poorly defined and located around POPA marker 2_0563, with no obvious candidate genes. Highly elevated Fe concentrations in grains were characteristic of IL146, but not found in ILs 118 to 121, each of which contain partially overlapping introgressions, with no promising candidate gene inside the small region that is possibly unique to IL146 (S2 Dataset). These observations highlight the future need for further genetic dissection of the introgressions of the most promising introgression lines.

Candidate genes could be identified for some additional QTL of interest. The ILs 117, 118 and 119 are jointly characterized by lowered Zn and Fe concentrations in the young leaf by comparison to Scarlett (S5 Table). Their partially overlapping introgressions on chromosome 4H are predicted to share three metal homeostasis candidate loci. The highest ranking BLASTP hit for one of these loci in *A*. *thaliana* was NRAMP2 (Natural Resistance-Associated Macrophage Protein 2). Other members of the Arabidopsis NRAMP family were shown to be essential for the remobilization of vacuolar Fe stores during seedling germination [47] and the mobilization of vacuolar Mn during Mn deficiency [48], for example. The two remaining candidate loci were annotated as being most similar to *AtZIFL1* (*Zinc Induced Facilitator-Like 1*). An Arabidopsis homologue of ZIFL1, ZIF1, mediates vacuolar sequestration of the low-molecular-weight chelator nicotianamine that is important in the homeostasis of both Zn and Fe [49,50].

An IL with multiple QTL effects was IL115, which exhibited elevated grain concentrations of both Cd (170% of Sc) and Zn (127% of Sc). The donor introgression contains eight loci with a putative functions in metal homeostasis, four of which were unique to IL115, and thus absent in ILs harboring overlapping introgressions which did not give rise to enhanced grain Zn or Cd concentrations (Table 2). Of these, based on the functions of previously characterized members of gene families, the most promising candidate genes for a role in grain Zn and Cd accumulation were similar to the Arabidopsis *Zrt/Irt-like Protein 2* (*ZIP2*) and *Metal Tolerance Protein 3* (*MTP3*) [45,51].

The concentration of Fe in the young leaf of ILs 104 and 157 was reduced to 82 and 64%, respectively, of that found in Scarlett. The segment of the target introgression that is shared by these two ILs is also present in IL102, in which, however, Fe concentrations in the young leaf were only 8% below Scarlett (no statistically significant difference). The overlapping portion of the target introgressions of IL104 and IL157 contains six loci with potential roles in metal homeostasis (Table 2). Among these, a gene homologous to *A*. *thaliana Zrt/Irt-like Protein 6* (*AtZIP6*) is a candidate for modulating tissue Fe concentrations [45]. In addition to their target introgressions, both IL104 and IL157 feature a non-target introgression on chromosome 2H. Whereas IL102 bears no introgression in this region of the barley genome, the non-target introgression in IL104 encompasses part of the non-target introgression of IL157. The combination of these findings highly support this shared region, in which, however, we could not identify any evident candidate genes. Interestingly, a different segment of the non-target introgression of IL157 overlaps partially with the target introgression of IL106, in which we detected a 75% reduction in young leaf Fe concentration in comparison to Scarlett (Table 2). Thus, the non-target introgression of IL157 might contain more than a single locus for which donor alleles contribute to lowered Fe concentration in the young leaf. The heavy metal transport/detoxification domain of the only metal homeostasis-related gene (best BLAST hit At5g66110) predicted in the region shared between the non-target introgression of IL157 and the target introgression of IL106 on chromosome 2H is unlikely to contribute to Fe homeostasis. Thus, using presently available genotyping information we could not identify any promising candidate genes in the introgressed non-target donor segment of IL157 on chromosome 2H. In summary, several candidate genes likely to influence barley metal homeostasis can now be targeted by a detailed functional analysis and comparison of the *Hordeum spontaneum* and the *H*. *vulgare* alleles.

Our QTL analysis also indicated that it is possible to manipulate the concentration of Zn or Fe in barley grains without affecting the accumulation of toxic non-target elements such as Cd. We were not successful at finding a QTL exclusively lowering grain Cd, with no effect at all on Zn concentration, although this was achieved in studies on rice [52,53]. In senesced flag leaf, we identified two QTL, in IL108 and IL147, for enhanced Cd accumulation (S4 Table).

In cereal species the use of ILs and subsequent genetic mapping revealed QTL [54,55] and individual genes [56–58,21] responsible for nutrient ion accumulation or tolerance of ion excess in crop plants. For grain Cd, Zn or Fe accumulation, several QTL studies were conducted in rice [59,60,52,53] and wheat [1,61–63]. Furthermore, a forward genetic screen identified *Os*NRAMP5 as a major protein that contributes to root Cd uptake in rice [64].

The *HvNAM-1* locus on chromosome 6H, in the interval between 40 and 96 cM (estimated position at 90 cM [65]), is orthologous to the wheat *NAM-B1* NAC-domain containing transcription factor gene, of which a loss-of-function allele, which is common in present-day cultivated wheat, results in reduced grain protein, Zn and Fe concentration [1]. The introgressions of ILs 128, 129, 149, 150 and 152 are known to contain the exotic allele at this locus, which was found to be associated with higher grain protein content in the same set of S42 ILs cultivated in the field [65]. Different from wheat, we detected significant effects for Fe or Zn concentrations only in the young leaf, but not in grains, of these barley ILs upon cultivation on a metal-contaminated soil (S5 Table).

The comparison of the elemental composition of the young leaf and the flag leaf suggested that overall, Cu, Fe and possibly Mn, were remobilized from the flag leaf during grain filling to a much larger extent than Zn (Figs 1 and 2). Based on the simplifying generalizations that the composition of the living flag leaf resembled that of the young leaf and that dry biomass of the flag leaf remained approximately constant during senescence, an estimate can be made that of the concentrations in the living flag leaf, an average of 27% Fe, 48% Cu and 16% Mn were remobilized towards the grains during grain filling. Earlier studies concluded that, in general, Cd accumulation in wheat and durum wheat grain is partially caused by remobilization from the flag leaf [66,67,1]. However, across ILs of this study, the relationship between element concentrations in grains, young leaf and flag leaf was complex (Fig 3). A significant trend was observed for higher grain Cd accumulation to be associated with lower young leaf *vs*. flag leaf concentration ratios. This indicated that, in addition to remobilization from the flag leaf, developmental stage-specific root uptake and translocation into the grain, possibly involving intervascular transfer [38,68], must make an important contribution to grain element concentrations (see also S2 Fig).

Fe concentrations were not strongly correlated with the concentrations of any other transition metal in any of the analyzed tissues. As a classical strategy II plant, barley exhibits phytosiderophore-mediated Fe acquisition. This may render the homeostasis of transition metals less dependent on Fe homeostasis than established in strategy I model plants [69]. In strategy I plants such as *A*. *thaliana*, Fe deficiency causes enhanced uptake rates for Cd^2+^ and other divalent transition metal cations *via* the non-selective iron uptake transport system IRT1. Instead, in this study between-metal correlations across barley ILs suggested that the pathways for Ca, Zn, Mn and Mg accumulation all contribute to Cd accumulation in the young leaf far more strongly than in grains. Importantly however, there was a pronounced correlation between grain Cd and Cu concentrations, suggesting that Cd shares with Cu an element of the movement pathway, for example a membrane transporter or binding partner, specifically during grain filling (see Fig 4). Among the responses of Arabidopsis to Cd exposure are altered Cu accumulation and transport processes, which contribute to basal Cd tolerance and depend on the *Squamosa Promoter-binding protein-Like 7* (*SPL7*) Cu deficiency response-regulatory transcription factor gene [70]. A similar link between Cd and Cu homeostasis might explain our observation in barley grains.

Recently, a differently designed study reported genome-wide association mapping-based QTL regions for grain Cd concentrations of 100 barley accessions cultivated in a non-contaminated field, as well as for root and shoot tissues of hydroponically cultivated seedlings [71].

### Conclusions

Our results suggested that barley restricted the entry of Cd, Ca and Mg into the grain through immobilization in the flag leaf. This study identified a number of introgressions from a wild barley accession that alter grain accumulation of Cd, Zn, Fe, Cu, Mn, Mg or Ca in the genetic background of an elite malting spring barley cultivar. Based on a total of 54 ILs, the identified introgressions are large. In the future, introgressions of the lines most divergent in grain Cd, Zn and Fe accumulation will be dissected genetically towards the identification of the functionally decisive genes through the analysis of sub-ILs, taking into account the candidate genes described here. For this purpose, existing high-resolution sub-IL populations [30] segregating for a candidate gene or target introgression will be subjected to genotyping-by-sequencing and examined with respect to their element accumulation properties. The identification of introgressions altering barley grain Fe, Zn or Cd accumulation, respectively, is a first step towards bio-fortification without simultaneously increasing Cd load in the human diet, despite the similarity of chemical properties and of bioaccumulation pathways of these micronutrient and toxic metal cations. This will help to decrease the Cd burden on human health originating from anthropogenically or geogenically Cd-enriched agricultural soils, which are widespread globally [72].

## Supporting Information

S1 Dataset Element concentrations in barley tissues (includes raw data, least-square means, SD, full model ANOVA table, and medians). (XLS)

S2 Dataset Candidate metal homeostasis genes in the exotic introgressions in ILs harboring QTL for Cd, Zn or Fe. (XLS)

S1 Fig Element accumulation in tissues of barley introgression lines by comparison to the recurrent parent Scarlett. (PDF)

S2 Fig Inter-organ relationships of element concentrations between the young leaf and mature grains. (PDF)

S1 Table Soil elemental composition. (PDF)

S2 Table Broad-sense heritability for element concentrations in three tissues. (PDF)

S3 Table Number of QTL detected for concentrations of each element per tissue. (PDF)

S4 Table QTL for Cd, Zn and Fe concentrations in the senesced flag leaf. (PDF)

S5 Table QTL for Cd, Zn and Fe concentrations in the young leaf. (PDF)
