# Supplementary material for: Quantitative Trait Loci and Inter-Organ Partitioning for Essential Metal and Toxic Analogue Accumulation in Barley
Source: PLoS One. 2016 Apr 14;11(4):e0153392. doi: 10.1371/journal.pone.0153392 (PMC4831800; doi:10.1371/journal.pone.0153392)
Supplement: S1 Fig — (PDF) [file pone.0153392.s003.pdf]

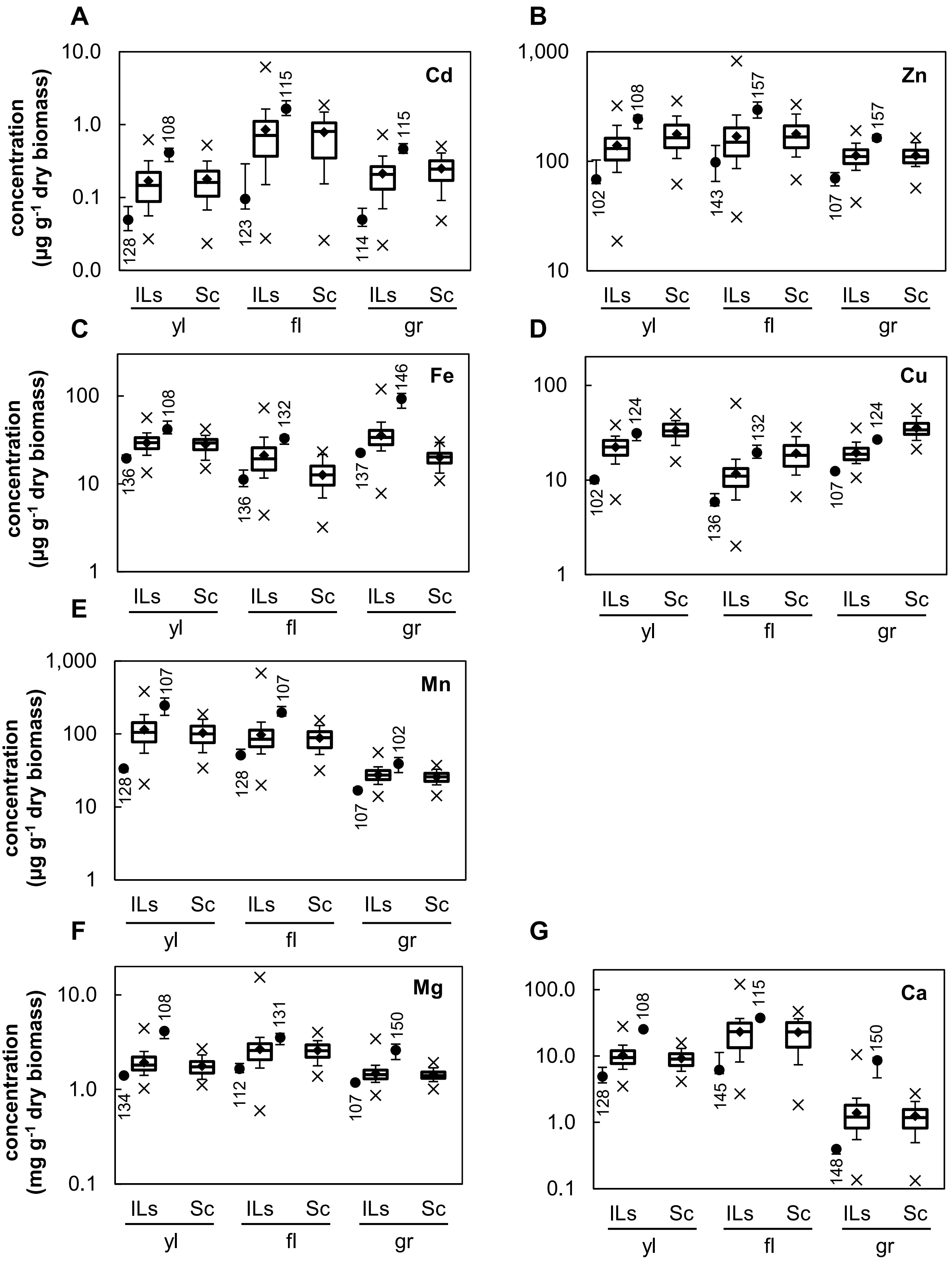

S1 Fig. Element accumulation in tissues of barley introgression lines by comparison to the recurrent parent Scarlett. Shown are summarizing boxplots of (A) Cd, (B) Zn, (C) Fe, (D) Cu, (E) Mn, (F) Mg, and (G) Ca concentrations measured by ICP-AES in young leaf (yl), flag leaf (fl) and grains (gr) of all measurements of 54 introgression lines (ILs) and all replicate individuals of the recurrent parent Scarlett (Sc). Diamonds show arithmetic means, boxes show

the 25-to-50-to-75 percentiles, whiskers show the 10-to-90 percentiles and 'x' symbols show the minimum and maximum measurements (n = 3 to 7 replicate individuals for each of 54 ILs; n = 144 for recurrent parent Scarlett). Circes to the left and right of the IL data show the median ± 25-to-75%-tile of the single IL exhibiting the lowest and highest median (n = 3 to 7), respectively, with the number identifying the corresponding IL given adjacent to each symbol. Note that medians and percentiles of individual ILs were calculated from raw data without accounting for positional effects, different from least-square means presented elsewhere. Plants were cultivated in a greenhouse on soil collected in a heavy-metal contaminated agricultural field, with harvest of yl at the five-leaf stage and harvest of fl and gr at maturity. Data are from the same experiment as shown in Fig 1.
