# Supplementary material for: Quantitative Trait Loci and Inter-Organ Partitioning for Essential Metal and Toxic Analogue Accumulation in Barley
Source: PLoS One. 2016 Apr 14;11(4):e0153392. doi: 10.1371/journal.pone.0153392 (PMC4831800; doi:10.1371/journal.pone.0153392)
Supplement: S2 Fig — (PDF) [file pone.0153392.s004.pdf]

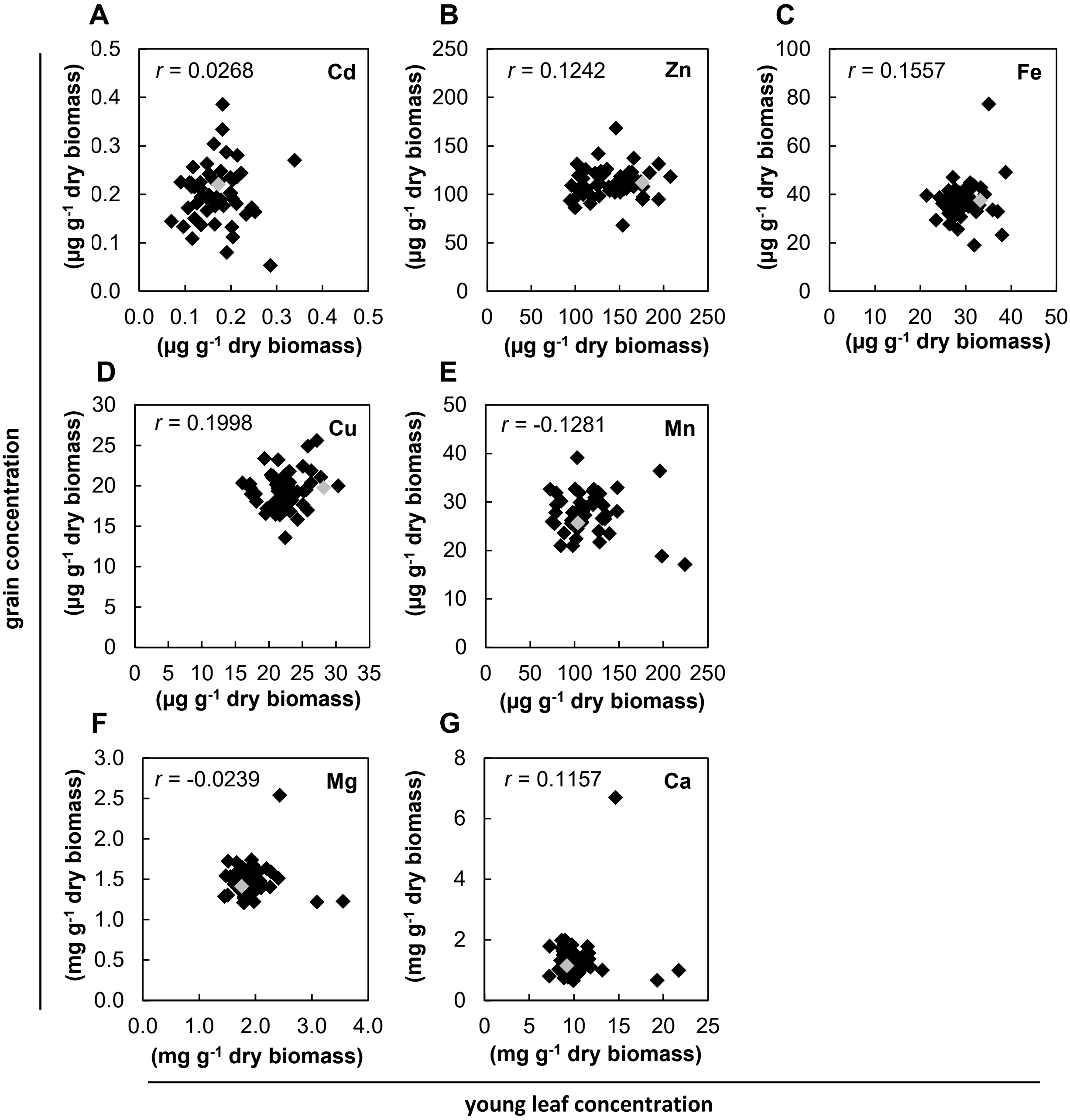

S2 Fig. Relationships of element concentrations between the young leaf and mature grains. Shown are scatterplots of concentrations in grains against concentrations in the young leaf for (A) Cd, (B) Zn, (C) Fe, (D) Cu, (E) Mn, (F) Mg, and (G) Ca. Each datapoint represents one introgression line (least-square mean, $n = 3$ to 7 per IL, for 54 ILs in total). Data for the recurrent parent Scarlett are shown in grey (least-square mean, $n = 144$). Pearson correlation coefficients (r) are given inside panels. Measurements were from the same experiment as shown in Fig 1.
