# Supplementary material for: Quantitative Trait Loci and Inter-Organ Partitioning for Essential Metal and Toxic Analogue Accumulation in Barley
Source: PLoS One. 2016 Apr 14;11(4):e0153392. doi: 10.1371/journal.pone.0153392 (PMC4831800; doi:10.1371/journal.pone.0153392)
Supplement: S1 Table — (PDF) [file pone.0153392.s005.pdf]

S1 Table. Soil elemental composition

Shown are arithmetic means of total and exchangeable element concentrations (± SD, *n* = 5) in the soil used for barley plant cultivation.

	Total (mg kg^{-1})	Exchangeable (mg kg^{-1})
Ag	4.3 ± 0.3	n.d.
Al	20,000 ± 2,000	17 ± 11
B	53 ± 3	0.4 ± 0.1
Ca	16,300 ± 500	2,300 ± 200
Cd	10 ± 1	0.08 ± 0.06
Co	27 ± 2	-
Cr	32 ± 3	0.04 ± 0.02
Cu	370 ± 30	2 ± 2
Fe	29,000 ± 1,500	31 ± 23
Hg	1.5 ± 0.1	-
K	5,100 ± 1,200	600 ± 300
Mg	3,700 ± 200	61 ± 8
Mn	2,800 ± 400	2.6 ± 1.7
Mo	1.8 ± 1.5	0.05 ± 0.03
Ni	30 ± 2	0.07 ± 0.03
P	1,500 ± 300	4.9 ± 2.3
Pb	3,600 ± 200	4.7 ± 3.1
S	1,400 ± 500	26 ± 7
Zn	3,400 ± 300	8.6 ± 4.9
