# Supplementary material for: Quantitative Trait Loci and Inter-Organ Partitioning for Essential Metal and Toxic Analogue Accumulation in Barley
Source: PLoS One. 2016 Apr 14;11(4):e0153392. doi: 10.1371/journal.pone.0153392 (PMC4831800; doi:10.1371/journal.pone.0153392)
Supplement: S2 Table — (PDF) [file pone.0153392.s006.pdf]

S2 Table. Broad-sense heritability for element concentrations in three tissues.

Shown is the broad-sense heritability (*H^2*) for the concentrations seven elements in three different tissues of barley in a population of 54 introgression lines.

**Trait***	**H^2 (%)**
Ca fl	74
Ca gr	86
Ca yl	91
Cd fl	71
Cd gr	84
Cd yl	76
Cu fl	60
Cu gr	79
Cu yl	85
Fe fl	61
Fe gr	91
Fe yl	88
Mg fl	15
Mg gr	83
Mg yl	87
Mn fl	70
Mn gr	86
Mn yl	87
Zn fl	61
Zn gr	83
Zn yl	81

* fl: flag leaf, yl: young leaf, gr: grains.
