# Supplementary material for: Quantitative Trait Loci and Inter-Organ Partitioning for Essential Metal and Toxic Analogue Accumulation in Barley
Source: PLoS One. 2016 Apr 14;11(4):e0153392. doi: 10.1371/journal.pone.0153392 (PMC4831800; doi:10.1371/journal.pone.0153392)
Supplement: S3 Table — (PDF) [file pone.0153392.s007.pdf]

S3 Table. Number of QTL detected for concentrations of each element per tissue.

Shown is the number of QTL identified for each element in each tissue, given by the level of statistical significance, including sub-totals (Sum) and totals.

	**Young leaf** (*P* <)				**Flag leaf** (*P* <)				**Grains** (*P* <)				
**Element**	0.001	0.01	0.05	Sum	0.001	0.01	0.05	Sum	0.001	0.01	0.05	Sum	**Total**
Cd			1	1	1		1	2		2		2	**5**
Zn			12	12		1		1	2	1		3	**16**
Fe	1	4	17	22					1	2		3	**25**
Cu	12	19	10	41							1	1	**42**
Mn	3			3	2			2	1	5	7	13	**18**
Mg	2	2	2	6			1	1	1		2	3	**10**
Ca	3		1	4		1		1	1			1	**6**
**Total**	**21**	**25**	**43**	**89**	**3**	**2**	**2**	**7**	**6**	**10**	**10**	**26**	**122**

P value, calculated by Dunnett's test followed by FDR-analysis
