# Supplementary material for: Quantitative Trait Loci and Inter-Organ Partitioning for Essential Metal and Toxic Analogue Accumulation in Barley
Source: PLoS One. 2016 Apr 14;11(4):e0153392. doi: 10.1371/journal.pone.0153392 (PMC4831800; doi:10.1371/journal.pone.0153392)
Supplement: S4 Table — (PDF) [file pone.0153392.s008.pdf]

S4 Table. QTL for Cd, Zn and Fe concentrations in the senesced flag leaf.

Magnitude of effect of each QTL is expressed as percentage of the concentration found in the parent genotype Scarlett (% of Sc).

IL Id.	Element	% of Sc	*P* value	Target introgression Chromosome	Target introgression Position (cM)[1]	Additional introgressions Position (cM)[1,2]
IL108	Cd	230	< 0.001	2H	34.31-104.81	2H 158.39-161.08 *h*
IL108	Zn	179	< 0.01	2H	34.31-104.81	2H 158.39-161.08 *h*
IL147	Cd	200	< 0.05	5H	145.57-154.37	3H 144.30 *h*

[1] Schmalenbach *et al*. 2011

[2] *h* following the genomic position indicates a hemizygous introgression.
