# Supplementary material for: Quantitative Trait Loci and Inter-Organ Partitioning for Essential Metal and Toxic Analogue Accumulation in Barley
Source: PLoS One. 2016 Apr 14;11(4):e0153392. doi: 10.1371/journal.pone.0153392 (PMC4831800; doi:10.1371/journal.pone.0153392)
Supplement: S5 Table — (PDF) [file pone.0153392.s009.pdf]

S5 Table. QTL for Cd, Zn and Fe concentrations in the young leaf.

Magnitude of effect of each QTL is expressed as percentage of the concentration found in the parent genotype Scarlett (% of Sc).

IL Id.	Element	% of Sc	*P* value	Target introgression Chromosome	Target introgression Position (cM)[1]	Additional introgressions Position (cM)[1,2]
IL102	Zn	59	< 0.05	1H	1.1-98.23	2H 90.54-98.35<br>3H 144.30-154.99 *h*
IL157	Fe	64	< 0.001		64.79-90.92	2H 20.11-22.35
IL104	Fe	82	< 0.05		70.78-78.03	2H 20.11<br>6H 36.93-38.38
IL143	Zn	64	< 0.05		130.68-173.49	5H 4.21<br>5H 133.74-134.85<br>6H 115.33-133.29<br>7H 49.53-51.93
IL142	Zn	62	< 0.05		188.5-205.07	-
IL106	Fe	76	< 0.01	2H	22.35-34.31	-
IL108	Cd	195	< 0.05		34.31-104.81	2H 158.39-161.08 *h*
IL111	Fe	83	< 0.05	3H	67.01-98.41	1H 74.40 *h*<br>1H 89.01<br>1H 173.49-199.04 *h*<br>2H 133.59-148.80
IL112	Fe	79	< 0.05		104.39-161.43	1H 190.63-202.26 *h*
IL140	Fe	79	< 0.05		154.99-253.73	-
IL116	Zn	62	< 0.05	4H	5.42-47.8	6H 35.85-38.48<br>6H 95.18-98.66
IL117	Zn	61	< 0.05		27.52-64.77	-
IL117	Fe	80	< 0.05		27.52-64.77	-
IL118	Zn	61	< 0.05		61.15-83.58	3H 144.30
IL118	Fe	71	< 0.01		61.15-83.58	3H 144.30
IL119	Zn	59	< 0.05		61.15-119.06	3H 144.30
IL119	Fe	82	< 0.05		61.15-119.06	3H 144.30
IL121	Fe	82	< 0.05		74.11-119.06	3H 185.12-211.25<br>6H 9.65 *h*
IL124	Fe	81	< 0.05		171.25-183.54	5H 268.55-274.24<br>7H 82.82
IL125	Fe	79	< 0.05	5H	104.73-154.37	3H 187.28-190.87 *h*
IL126	Fe	81	< 0.05		145.57-200.12	-
IL127	Zn	57	< 0.05		231.75-276.77	-
IL148	Fe	76	< 0.05	6H	3.28-10.73	3H 173.82-211.25<br>4H 74.11 *h*
IL128	Zn	55	< 0.05		71.39-132.23	1H 190.63-199.04
IL128	Fe	75	< 0.01		71.39-132.23	1H 190.63-199.04
IL149	Zn	58	< 0.05		71.39-82.43	6H 107.26-111.56
IL149	Fe	73	< 0.01		71.39-82.43	6H 107.26-111.56
IL129	Fe	82	< 0.05		73.9-133.47	-
IL150	Fe	82	< 0.05		73.9-82.43	6H 137.78-140.00

IL Id.	Element	% of Sc	*P* value	Target introgression Chromosome	Target introgression Position (cM)[1]	Additional introgressions Position (cM)[1,2]
IL122	Fe	81	< 0.05		180.69-208.13	4H 133.98.150.34 *h*<br>6H 137.78-140.00<br>7H 0.00-0.87
IL134	Fe	83	< 0.05	7H	51.93-107.44	3H 204.48-211.25 *h*
IL135	Fe	80	< 0.05		101.23-152.29	-
IL137	Fe	80	< 0.05		134.43-193.89	2H 83.61-90.54<br>2H 110.84-120.83<br>3H 185.12 – 211.25<br>4H 39.48 – 47.80
IL136	Zn	54	< 0.05		134.43-152.29	2H 110.84<br>4H 39.48-47.80
IL138	Zn	54	< 0.05		176.37-229.66	1H 106.61-143.47 *h*

[1] Schmalenbach *et al*. 2011

[2] *h* following the genomic position indicates a hemizygous introgression.
